# A Small Molecule Selected from a DNA‐Encoded Library of Natural Products That Binds to TNF‐*α* and Attenuates Inflammation In Vivo

**DOI:** 10.1002/advs.202201258

**Published:** 2022-05-21

**Authors:** Shuyue Wang, Xiaojie Shi, Jie Li, Qianping Huang, Qun Ji, Ying Yao, Tao Wang, Lili Liu, Min Ye, Yun Deng, Peixiang Ma, Hongtao Xu, Guang Yang

**Affiliations:** ^1^ Shanghai Institute for Advanced Immunochemical Studies ShanghaiTech University Shanghai 201210 P. R. China; ^2^ School of Life Science and Technology ShanghaiTech University Shanghai 201210 P. R. China; ^3^ Institute of Biochemistry and Cell Biology Shanghai Institutes for Biological Sciences Chinese Academy of Sciences Shanghai 200031 P. R. China; ^4^ University of Chinese Academy of Sciences Beijing 100049 P. R. China; ^5^ State Key Laboratory of Natural and Biomimetic Drugs School of Pharmaceutical Sciences Peking University Beijing 100871 P. R. China; ^6^ State Key Laboratory of Southwestern Chinese Medicine Resources School of Pharmacy Chengdu University of Traditional Chinese Medicine Chengdu Sichuan 611137 P. R. China; ^7^ Shanghai Key Laboratory of Orthopedic Implants Department of Orthopedic Surgery Shanghai Ninth People's Hospital Shanghai Jiao Tong University School of Medicine Shanghai 200011 P. R. China

**Keywords:** DNA‐encoded library, flavonoid, inflammation, kaempferol, tumor necrosis factor *α*

## Abstract

Tumor necrosis factor *α* (TNF‐*α*) inhibitors have shown great success in the treatment of autoimmune diseases. However, to date, approved drugs targeting TNF‐*α* are restricted to biological macromolecules, largely due to the difficulties in using small molecules for pharmaceutical intervention of protein–protein interactions. Herein the power of a natural product‐enriched DNA‐encoded library (*n*DEL) is exploited to identify small molecules that interfere with the protein–protein interaction between TNF‐*α* and the cognate receptor. Initially, to select molecules capable of binding to TNF‐*α* , “late‐stage” DNA modification method is applied to construct an *n*DEL library consisted of 400 sterically diverse natural products and pharmaceutically active chemicals. Several natural products, including kaempferol, identified not only show direct interaction with TNF‐*α*, but also lead to the blockage of TNF‐*α*/TNFR1 interaction. Significantly, kaempferol attenuates the TNF‐*α* signaling in cells and reduces the 12‐O‐tetradecanoylphorbol‐13‐acetateinduced ear inflammation in mice. Structure‐activity‐relationship analyses demonstrate the importance of substitution groups at C‐3, C‐7, and C‐4' of kaempferol. The *n*DEL hit, kaempferol, represents a novel chemical scaffold capable of specifically recognizing TNF‐*α* and blocking its signal transduction, a promising starting point for the development of a small molecule TNF‐*α* inhibitor for use in the clinical setting.

## Graphical abstract

The natural product‐enriched DNA‐encoded library (*n*DEL) approach, merging natural products with DEL, is applied in a reverse pharmacology study targeting tumor necrosis factor *α* (TNF‐*α*), a key cytokine in inflammation. Several natural products, particularly kaempferol, a flavonoid, has been identified to recognize and attenuate the TNF‐*α* signaling in cells as that of adalimumab, and reduce 12‐O‐tetradecanoylphorbol‐13‐acetate‐induced ear inflammation in mice.

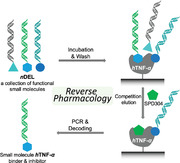

## 1 Introduction

DNA‐encoded chemical libraries (DELs) link the power of chemistry and genetics and have revolutionized drug discovery.^[^ ^1^ ^]^ DELs allows the simultaneous screening of an enormously large number of chemicals against various targets, thus greatly accelerating the discovery process in the search for new drugs. However, this major advancement does itself bring new challenges in terms of library expansion and DEL selection. The ability to select functional molecules using DEL technology is undoubtedly dependent upon both the total number and the scaffold diversity of members comprising the initial DEL library.^[^ ^2^ ^]^ Academic laboratories and pharmaceutical corporations are continually developing new DNA‐compatible chemical reactions, leading to a significant increase in the number of chemical classes that can now be included in DELs.^[^ ^3^ ^]^ At the same time, new selection strategies, especially for membrane‐bound protein targets, are being developed.^[^ ^4^ ^]^ Consequently, more and more DEL‐derived new drug candidates are entering clinical development.^[^ ^5^ ^]^

Tumor necrosis factor *α* (TNF‐*α*), a crucial regulator in the immune response, has been the target of multiple approved antibody drugs such as adalimumab, golimumab, and certolizumab.^[^ ^6^ ^]^ TNF‐*α* antagonism has shown therapeutic efficacy for several autoimmune diseases such as rheumatoid arthritis (RA),^[^ ^7^ ^]^ psoriatic arthritis, multiple sclerosis (MS), and Crohn's disease.^[^ ^8^ ^]^ Nevertheless, only 60–70% of patients achieve a long‐term clinical response. In the remaining patients, the poor response to antibody therapies is thought to be at least partially due to the immunogenicity of these biological molecules.^[^ ^9^ ^]^ This, in combination with other shortcomings of bio‐macromolecular drugs, such as high cost (for chronic usage), poor stability,^[^ ^10^ ^]^ and the requirement for parenteral administration, has prompted increasing interest in the development of orally bioavailable small molecule inhibitors targeting TNF‐*α*.

Over the course of evolution, natural products, including the active ingredients in Traditional Chinese Medicines (TCMs), have developed with highly diverse and complicated chemical scaffolds. These molecules have been extensively studied and used in the clinical treatment of disease for thousands of years.^[^ ^11^ ^]^ Many highly effective drugs are, in fact, derived from natural products, that is, Taxol^[^ ^12^ ^]^ and artemisinin.^[^ ^13^ ^]^ However, due to their polypharmacology, the mechanisms of action for most therapeutic natural products remain largely unclear. Although the polypharmacology of natural products hinders their further pharmaceutical development, it makes them ideal tools for chemogenomic studies.^[^ ^14^ ^]^ By merging natural products with DEL technology, one is able to fully leverage the power of natural selection in evolution. We developed a late‐stage DNA‐coding method that allowed us to generate a natural product‐enriched DEL library (*n*DEL), in which molecules with biological activities are DNA‐labeled and can be profiled via affinity panning.^[^ ^15^ ^]^

In this study, we used the *n*DEL, an approach that combines the advantages of both structural diversity and polypharmacological function, to probe small molecule binding pockets on TNF‐*α*. A natural product, kaempferol, was selected and shown to not only bind TNF‐*α* but also disrupt the interaction between TNF‐*α* and its cognate receptor TNFR1. It was capable of potent inhibition of both TNF‐*α*‐induced cell death and the corresponding signaling pathways in L929 cells. Furthermore, studies using a murine ear model demonstrated that kaempferol reduced inflammation in vivo.

## 2 Results

### 2.1 Affinity Panning and Competitive Enrichment of *n*DEL Molecules versus TNF‐*α*

**Figure 1:**
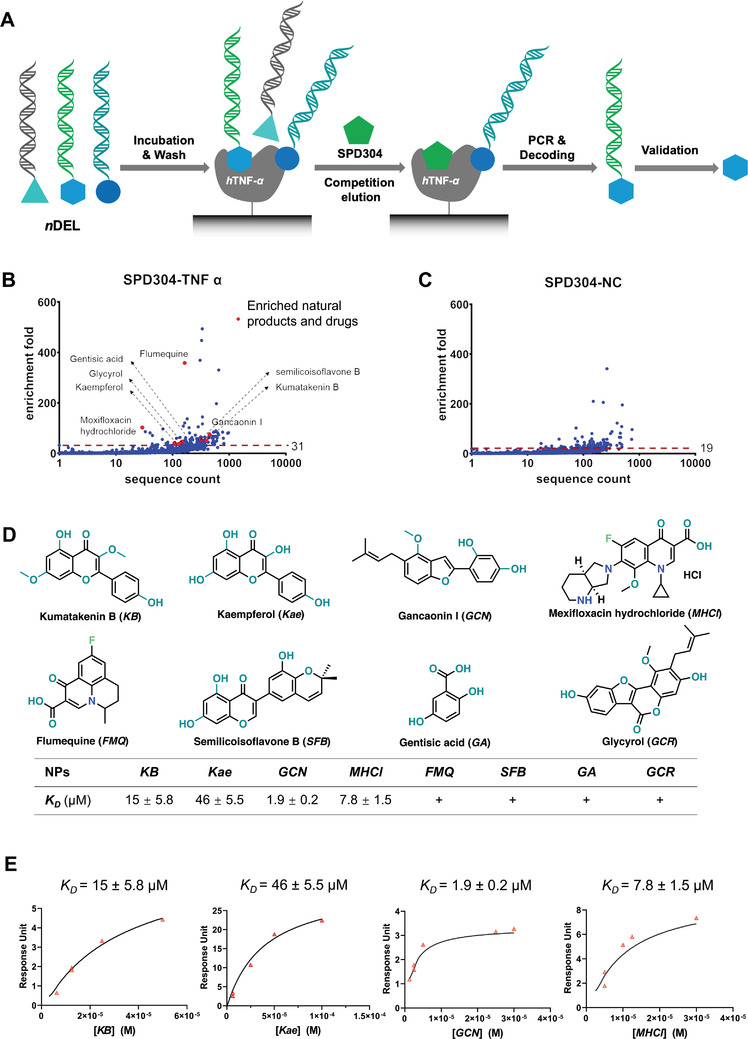
Library panning and hit identification of *n*DELs targeting *h*TNF‐*α*. A) Workflow of competitive panning of *n*DEL library. SPD304, a known small molecule binder to TNF‐*α*, serves as the competition eluent (red pentagon). B,C) Fingerprint plots for the *n*DEL screenings enriched by *h*TNF‐*α* coated or empty magbeads, respectively, in which *y*‐axis represents enrichment‐folds, while *x*‐axis represents sequence counts; red dashed lines are the cut‐off values for hits selection and red dots represent enriched *n*DELs. D) Chemical structures (upper panel) and summary table (lower panel) of confirmed *n*DEL hits, in which four compounds, including kumatakenin B (*KB*), kaempferol (*Kae*), gancaonin I (*GCN*), and moxifloxacin (*MHCl*), showed measurable *K*_D,app_ values, and the other four compounds, including flumequine (*FMQ*), semilicoisoflavone B (*SFB*), gentisic acid (*GA*), and glycyrol (*GCR*), showed weak binding. The “+” sign in the lower panel table represents a weak binding signal in SPR sensorgram that is too weak to quantitate. E) Apparent *K*_D,app_ values of the four potent *n*DEL hits. All results are shown as means ± SD.

Recombinant human TNF‐*α* (*h*TNF‐*α*) was purified with Ni‐NTA and size‐exclusion chromatography (SEC) (Figure S1A–C, Supporting Information). Analytical SEC was carried out using authentic BSA (MW: 66.4 kDa) and lysozyme (MW: 17.9 kDa) as retention volume markers. The recombinant his‐tagged *h*TNF‐*α* appeared to be a trimer (approx. MW: 55.2 kDa) with an SEC retention volume close to that of BSA (MW: 66.4 kDa) (Figure S2, Supporting Information), consistent with previous reports.^[^ ^16^ ^]^ The purified protein was biotinylated via lysine conjugation and then immobilized on streptavidin beads. An *n*DEL library containing 400 natural products and pharmaceutical active small molecules in addition to 10^4^ combinatorial chemicals was constructed by the late‐stage DNA annotation method reported previously (Figure S3 and Experimental Methods, Supporting Information).^[^ ^15a^ ^]^ As shown in **Figure** **1**A, the recombinant *h*TNF‐*α*‐coated beads were mixed with the *n*DEL library, incubated, and washed to remove any non‐specific binders. Bound molecules were then eluted using SPD304, a known small molecule TNF‐*α* inhibitor.^[^ ^17^ ^]^ The eluent was subjected to DNA‐sequencing for structure decoding. The panning fingerprints of the eluted molecules were plotted as enrichment‐fold versus normalized sequencing counts (Figure 1B), and compared with that of the negative control (Figure 1C). Seven natural products and one known drug molecule showed significant enrichment (Figure 1D,E) and were selected for further study.

### 2.2 Affinity Measurement of *n*DEL Hits Bound to TNF‐*α*

To confirm the binding of the above hit molecules to *h*TNF‐*α*, surface‐plasma‐resonance (SPR) was used to measure the affinity of the interaction. A CM5 chip was used with a running buffer consisting of 1% DMSO. Recombinant *h*TNF‐*α* first flowed through and immobilized on the CM5 chip via amine coupling to form a uniform single molecular layer of *h*TNF‐*α* on the chip surface. Of the eight *n*DEL hits, all showed some degree of interaction with the *h*TNF‐*α* coating surface (Figure 1D). Four of these, three natural products and one known antibiotic displayed measurable binding affinities. The two flavonoid natural products, kaempferol (*Kae*) and kumatakenin B (*KB*), showed similar *K*_D_ values of 45 ± 5.5 and 15 ± 5.8 µm, respectively; whereas, the natural product, gancaonin I (*GCN*), and the antibiotic, moxifloxacin hydrochloride (*MHCl*), showed more potent binding with *K*_D_ values of 1.9 ± 0.2 and 7.8 ± 1.5 µm, respectively (Figure 1D,E and Figure S4, Supporting Information).

### 2.3 Structure–Activity Relationship of Kaempferol Analogues

**Figure 2:**
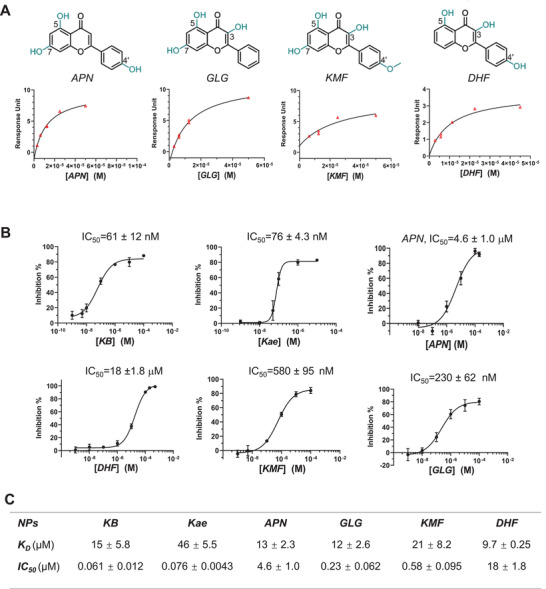
SAR analysis of flavonoid analogues. A) Chemical structures (upper panel) and *K*_D,app_ measurements (lower panel) of apigenin (*APN*), galangin (*GLG*), kaempferide (*KMF*), and 4',5‐dihydroxyflavone (*DHF*). B) Competitive affinity measurement of flavonoids to *h*TNF‐*α* in the presence of *h*TNFR1‐ECD‐HRP by ELISA assay (*n* = 6). C) Summary table of apparent binding affinity of flavonoids to *h*TNF‐*α* in the absence (*K*_D,app_) and presence (*IC*_50_) of *h*TNFR1‐ECD‐HRP. All results are shown as means ± SD.

To further explore the relationship between the structure and affinity of flavonoids to TNF‐*α*, we tested four additional flavonoid analogs of kaempferol that are commercially available and contain different numbers of hydroxyl or methoxyl substitution groups (**Figure** **2**A and Figure S4, Supporting Information) using SPR. As shown in Figure 2B, similar to *Kae* and *KB*, apigenin (*APN*), galangin (*GLG*), kaempferide (*KMF*), and 4',5‐dihydroxyflavone (*DHF*) all interacted with the recombinant TNF‐*α*. Compared to *Kae*, removing any of the —OH group at the C3 (e.g., *APN*), C7 (e.g., *DHF*), or C4’ (e.g., *GLG*) position resulted in improved binding to *h*TNF‐*α*. In contrast, methylation at C3 and C7 (e.g., *KB*) or at C4’ (e.g., *KMF*) had little effect on the binding affinity (Figure 2C).

### 2.4 Blocking of Protein–Protein Interaction between TNF‐*α* and TNFR1

**Figure 3:**
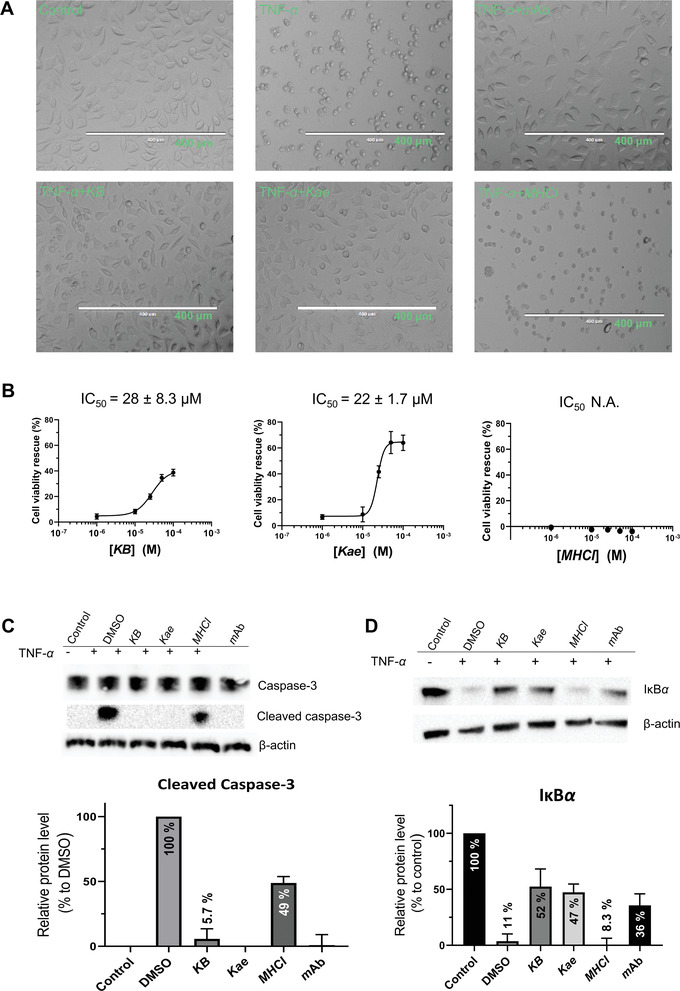
Effects of *n*DEL hits on cellular TNF‐*α* signaling. A) Morphology images of L929 cells upon treatment with actinomycin D (Control), *h*TNF‐*α* (TNF‐*α*), *h*TNF‐*α* and adalimumab (TNF‐*α*+mAb), *h*TNF‐*α* and *KB* (TNF‐*α*+*KB*), *h*TNF‐*α* and *Kae* (TNF‐*α*+*Kae*), and *h*TNF‐*α* and *MHCl* (TNF‐*α*+*MHCl*), respectively. B) Dose‐dependent rescue of *h*TNF‐*α* induced cell death of L929 by *KB*, *Kae*, and *MHCl* (*n* = 6). C) Western‐blot analyses of cellular caspase‐3 and cleaved‐caspase‐3. D) Western‐blot analyses of cellular I*κ*B*α*. Relative intensity of cleaved caspase‐3 for each testing compound was quantitated by normalizing against band intensity of the internal *β*‐actin and subtracting band intensity of background. Results are shown as means ± SD (*n* = 6).

To further explore if binding to TNF‐*α* by compounds selected from our *n*DEL inhibited the binding of TNF‐*α* to its cognate receptor, TNF‐*α* receptor 1 (TNFR1), a competitive affinity assay was performed using ELISA. First, the cognate ligand‐receptor interaction was established in a 96‐well microtiter plate using recombinant *h*TNF‐*α* and HRP‐tagged *h*TNFR1 extracellular domain (*h*TNFR1‐ECD‐HRP) (Figure S1D,E, Supporting Information). The apparent *EC*_50_ value of *h*TNFR1‐ECD‐HRP to *h*TNF‐*α* was determined to be 4.6 ± 0.3 nm (Figure S5A, Supporting Information). All compounds to be tested were then examined and shown to have a minimal disturbance of absorption at wavelength 450 nm under the assay conditions. The competitive assay was carried out by pre‐mixing *h*TNFR1‐ECD‐HRP (4.6 nm) with test compounds at various concentrations in a 100 µL assay solution, followed by incubation in a 96‐well microtiter plate coated with *h*TNF‐*α* . In the presence of TNFR1, the non‐flavonoid compounds, *MHCl* and *GCN*, showed either no or greatly reduced (>5000‐fold less) binding potency, respectively (Figure S6, Supporting Information), which was in line with the lack of efficacy for these molecules seen in later cellular functional assays (**Figure** **3**A,B). In contrast, the flavonoid compounds, *Kae* and *KB*, showed nearly three orders of magnitude enhancement in binding potency in the presence of TNFR1, with apparent *IC*_50_ values of 76 ± 4.3 and 61 ± 12 nm, respectively (Figure 2B,C). Compared to *Kae*, methylation of the –OH group at C3 and C7 (e.g., *KB*) did not affect the potency of competitive binding, whereas removal of either the –OH at C3 (e.g., *APN*) or at C7 (e.g., *DHF*) resulted in a potency decrease of greater than two orders of magnitude (Figure 2B,C). Moreover, methylation (e.g., *KMF*) or removal (e.g., *GLG*) of the –OH substitution at C4’ both resulted in a modest 2–5 fold reduction in binding potency (Figure 2B,C). Flavonoids appeared to display a very different structure–activity relationship (SAR) trend of binding to TNF‐*α* with or without the presence of TNFR1.

### 2.5 *Kae* Rescued TNF‐*α*‐Induced Cell Death by Blockage of TNF‐*α* Cell Signaling

TNF‐*α* is a key molecule in the inflammatory response. It is a multifunctional, pro‐inflammatory cytokine involved in various physiological and pathological processes, including cell proliferation, differentiation, and apoptosis, as well as immune modulation and the induction of inflammation.^[^ ^18^ ^]^ In the following experiment, TNF‐*α‐*induced apoptosis was measured using a proliferation assay in the L929 cell line that overexpresses TNFR1 (Figure S5B, Supporting Information). Adalimumab, an anti‐TNF‐*α* antibody known to inhibit TNF‐*α*‐induced apoptosis,^[^ ^19^ ^]^ was used as a positive control. Initially, we showed that the *n*DEL hit *GCN* showed apparent intrinsic cytotoxicity to the L929 cell line (Figure S7, Supporting Information) and was excluded from the following rescue experiment. The protective effect of the two flavonoids, *Kae* and *KB*, and the antibiotic *MHCl* on TNF‐*α*‐induced cell death of L929 was examined and compared to that of adalimumab. As expected, *MHCl*, which only binds to TNF‐*α* in the absence of *h*TNFR1‐ECD, showed no apparent protection at 100 *μ* m; whereas the flavonoids, that bind to TNF‐*α* in both the presence and absence of *h*TNFR1‐ECD, displayed a strong protective effect similar to that of adalimumab (Figure 3A). Furthermore, *KB* and *Kae*, the two flavonoids showing similar potency in the previously‐described *h*TNFR1‐ECD competition assay, rescued the TNF‐*α*‐induced cell death of L929 in a dose‐dependent manner, with compatible apparent *IC*_50_ values of 28 ± 8.3 and 22 ± 1.7 µm, respectively (Figure 3B).

TNF‐*α* cell signaling via TNFR1 orchestrates an intricate cellular network of signal pathways which can lead to two paradoxical effects; a) stimulation of cell survival and expression of pro‐inflammatory genes or b) apoptosis and cell death. TNF‐*α‐*induced activation of the caspase cascade represents a hallmark of TNF‐*α* signaling leading to programmed cell death (apoptosis).^[^ ^20^ ^]^ We further investigated if *KB* and *Kae* rescued cells from TNF‐*α*‐induced cell death by inhibiting the caspase cascade activation in L929 cells. The intrinsic effects of the tested compounds on the caspase cascade were first ruled out (Figure S8A, Supporting Information). As shown in Figure 3C, at 50 *μ* m, *KB* and *Kae*, like adalimumab, blocked completely the TNF‐*α*‐induced cleavage of caspase‐3. *MHCl*, on the other hand, showed only partial inhibition (56% inhibition) of caspase cascade activation at 100 *μ* m.

Induction of mitogenesis is another hallmark of TNF‐*α* signaling and is exerted by the activation of key transcription factors such as NF‐*κ*B and AP1. NF‐*κ*B activation results in the degradation of I*κ*B*α* protein, a negative regulator of NF‐*κ*B, in cells.^[^ ^21^ ^]^ We examined the ability of *Kae* and *KB* to inhibit TNF*‐α‐*induced NF‐*κ*B activation by monitoring I*κ*B*α* degradation in L929 cells. The intrinsic effects of the tested compounds on I*κ*B*α* degradation were first ruled out (Figure S8B, Supporting Information). As shown in Figure 3D, both *Kae* and *KB*, at 50 *μ* m, inhibited ≈45% TNF*‐α‐*induced I*κ*B‐*α* degradation in L929 cells. Adalimumab also showed a similar partial inhibition at a saturated concentration (10 nm). Moreover, when compared to the background control, *MHCl* showed no inhibition of TNF‐*α‐*induced I*κ*B*α* degradation at 100 *μ* m.

### 2.6 *Kae* Attenuated TPA‐Induced Ear Inflammation in Mice

**Figure 4:**
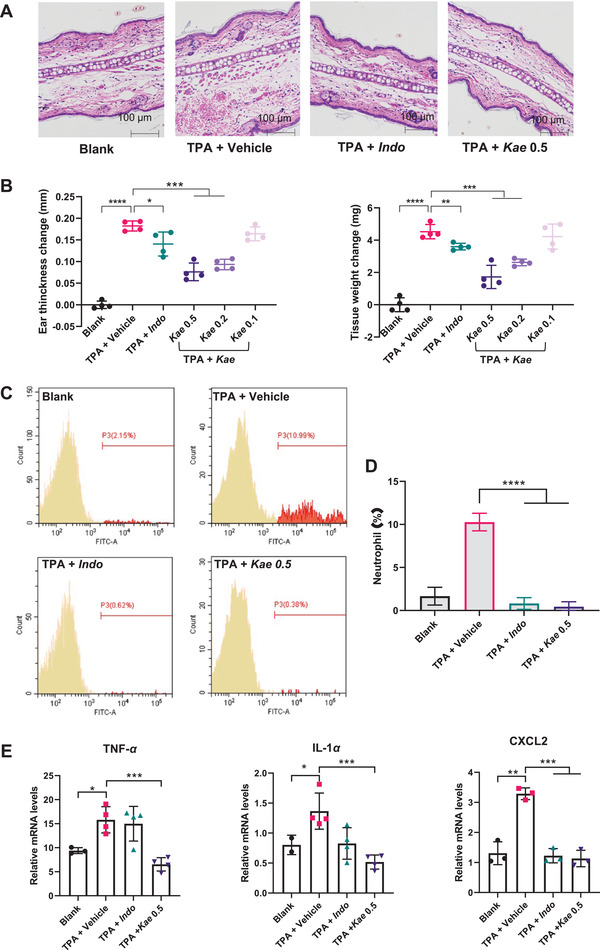
Anti‐inflammatory effect of *Kae* in TPA induced skin edema mouse model. A) Representative images (200 × magnification) of H&E stained mice ear sections from four cohort groups, in which *Indo* represents the positive control, indomethacin; Blank represents the untreated group (without TPA and compound treatment); TPA+Vehicle represents the group treated with 2.4 µg TPA and neat DMSO (vehicle); TPA+*Indo* represents the group with 2.4 µg TPA stimulation and 0.5 mg *Indo* treatment; and TPA+*Kae* 0.5 represents the group with 2.4 µg TPA stimulation and 0.5 mg *Kae* treatment. B) Dose‐dependent efficacy of *Kae* against TPA‐induced ear edema in mice ear (*n* = 4). Normalized ear thickness and ear weight of mice in different cohort groups are recorded and compared. For each mouse, normalization was carried out by subtracting the thickness and weight of the other untreated mouse ear from those of the treated ear. *Kae* 0.5, *Kae* 0.2, and *Kae* 0.1 represent different *Kae* dosages of 0.5, 0.2, and 0.1 mg, respectively. C) Representative FACS histograms. D) Quantitative FACS results showing the comparison of neutrophil amounts in peripheral blood of experimental mice (*n* = 4). E) mRNA expression of TNF‐*α*, IL‐1*α*, and CXCL2 in experimental mice ear determined by RT‐PCR (*n* = 4). All results were shown as means ± SD. *p*‐values are calculated using one‐way ANOVA with Bonferroni correction, **p < 0.01, ***p < 0.001, ****p < 0.0001.

To further investigate the anti‐inflammatory effect of the flavonoids *Kae* and *KB*, an in vivo, phorbol ester 12‐O‐tetradecanoylphorbol‐13‐acetate (TPA)‐induced skin edema model was established in C57BL/6 mice. TNF‐*α* is a key factor involved in TPA‐induced dermatitis, especially at the stage where dermal edema is significant.^[^ ^22^ ^]^ Topical application of TPA has been shown to induce TNF‐*α* expression at the application site.^[^ ^22a^ ^]^ Exposure of a mouse ear to 2.4 µg TPA (in neat DMSO) for 30 min led to a significant increase in ear thickness and tissue weight (**Figure** **4**A). Indomethacin (*Indo*), a COX inhibitor, was used as a positive control for anti‐inflammation.^[^ ^23^ ^]^ In a typical experiment, *Indo* (0.5 mg in neat DMSO) or the test compound, *Kae* (0.1, 0.2, 0.5 mg in neat DMSO), was applied topically to the TPA‐treated area at 30 min and 3.5 h after TPA exposure. 3 h following the second application of *Indo* or *Kae*, tissue samples were collected for analysis. In experimental mice (four mice/cohort group), the contralateral ear served as an internal background control and was not treated with either TPA or test compounds. As shown in Figure 4A, TPA treatment resulted in a significant swelling of the treated ear, while the contralateral ear showed no effect. This TPA‐induced inflammation was greatly attenuated following treatment with either 0.5 mg *Indo* or 0.5 mg *Kae* (Figure 4A). Both ear thickness and tissue weight in *Kae*‐treated mice were reduced by levels comparable to those achieved in *Indo*‐treated mice (Figure 4B), indicating a strong anti‐inflammatory effect for *Kae*. Furthermore, *Kae* was shown to reduce mouse ear swelling in a concentration‐dependent manner (Figure 4B).

To further understand the underlying mechanism of the observed *Kae* effect, peripheral neutrophil levels and tissue mRNA levels of pro‐inflammatory cytokines (TNF‐*α*, IL‐1*α*, and CXCL2) were measured, respectively. TPA‐treated mice had an increased level of neutrophils in their peripheral blood, as well as increased mRNA expression of TNF‐*α*, IL‐1*α*, and CXCL2 in the tissues of the treated ear. Application of *Kae* following TPA exposure completely suppressed the increase in peripheral neutrophils and the increase in mRNA expression of all three cytokines (Figure 4C–E). In contrast, although TPA‐treated mice receiving subsequent treatment with *Indo* showed complete suppression of the increase in peripheral neutrophils, only mRNA expression of CXCL2 and IL‐1*α*, but not TNF‐*α*, was suppressed in the treated tissue samples (Figure 4C–E). This is consistent with the different anti‐inflammatory mechanisms for the observed effects of kaempferol and indomethacin.

## 3 Discussion

Polypharmacological profiling of multiple targets is an important yet challenging endeavor in drug discovery. In an effort to dissect the underlining mechanisms of diseases and other biological phenotypes, the chemogenomic approach has been developed to link chemical structures with bioactivity. Current chemogenomic research relies on existing knowledge databases and is limited to a few disease areas, that is, CNS, drug abuse (DA), and cancer.^[^ ^14a^ ^]^ Natural products‐enriched DEL library, acting as a molecular probe of polypharmacology, enables the chemogenomic approach readily applicable to a wide range of research efforts.

DEL approach was successfully applied in an attempt to identify novel small molecule inhibitors of TNF‐*α* before.^[^ ^24^ ^]^ In the current study, we took advantage of the polypharmacological characteristic of an *n*DEL library consisting of 400 biologically active compounds (Table S4, Supporting Information) to map potential small molecule binding pockets on TNF‐*α*. Kaempferol, a flavonoid natural product, was identified as one of the compounds capable of direct binding to the *h*TNF‐*α*. It was shown to inhibit TNF‐*α*‐induced signaling transduction in L929 cells and attenuate ear inflammation in an acute mouse model. Kaempferol, extracted mainly from *Kaempferol galanga L*, is widely found in many edible plants and herbal medicines,^[^ ^25^ ^]^ and is known for its anti‐inflammatory and anti‐oxidative effects.^[^ ^26^ ^]^ Significantly, kaempferol has been shown to attenuate the severity of arthritis in a collagen‐induced arthritis (CIA) mouse model.^[^ ^27^ ^]^ In addition, it has a high safety profile in terms of both liver and renal side effects. Our current study established that kaempferol can exert its anti‐inflammatory effect via interruption of TNF‐*α* cell signaling similar to Humira (adalimumab).

Polypharmacology associated with chemical drugs is a key factor for their adverse effects in clinical application. *MHCl*, a quinolone antibiotic, is known to potently inhibit the enzymatic activities of bacterial DNA gyrase and topoisomerase IV leading to strong antimicrobial activity. Its off‐target inhibition of mammalian topoisomerase II at high concentration (>100 *μ* m), on the other hand, is believed as one of the important factors causing various adverse events of *MHCl* such as cardiac, hepatic, and cutaneous toxicities.^[^ ^28^ ^]^ Our observation that *MHCl* is a potent TNF‐*α* binder (apparent *K*_D_ = 7.8 *μ* m) provides new insight into the inflammatory nature of this molecule. Interestingly, our study showed that the binding of *MHCl* to TNF‐*α* did not interfere with the TNF‐*α*‐TNFR1 signaling. *MHCl* showed only partial inhibition of TNF‐*α* induced caspase cascade with no inhibition of TNF‐*α* induced NF*κ*B activation (Figure 3C,D).

To date, several small molecules that directly interact with TNF‐*α* have been identified, including SPD304, a potent but cytotoxic chemical;^[^ ^17^ ^]^ C87, a compound blocking TNF‐*α*‐induced signaling but not TNF‐*α* binding to TNFR1;^[^ ^29^ ^]^ and benpyrine, a natural product molecule with weak potency.^[^ ^30^ ^]^ He et al. proposed a binding mode of SPD304 in TNF‐*α* regulation, in which an SPD304 molecule displaced a subunit from the native trimeric TNF‐*α* complex to form a complex with a dimeric TNF‐*α*.^[^ ^17^ ^]^ The binding mode of kaempferol with the TNF‐*α* trimer is currently under investigation using cryo‐EM and X‐ray crystallography. Our affinity study of *n*DEL molecules to TNF‐*α* in the presence and absence of TNFR1 demonstrated a synergistic interaction between flavonoids and TNFR1 that is correlated to the cellular TNF‐*α*/TNFR1 signaling pathway. Our preliminary analyses of the chemical structure and TNF‐*α* function suggested that the steric interactions at C‐3 and C‐7 and the H‐bonding at C‐4 of kaempferol appeared to be essential. Our current study demonstrated the power of combining natural products with DEL technology in polypharmacological research, an emerging paradigm in next‐generation drug discovery and development.

## 4 Conclusion

In this study, we demonstrated that DEL technology, in combination with natural products and functional small molecules (*n*DEL approach), could serve as an invaluable polypharmacological probe in chemogenomic research. As a pilot study for proof‐of‐concept, TNF‐*α*, a key cytokine in inflammatory responses, was used as the target protein. A natural‐product small molecule binder of TNF‐*α*, *Kae*, was identified by *n*DEL panning and showed impressive inhibitory efficacies on TNF‐*α* induced cell signaling both in vitro and in vivo similar to that of the known anti‐inflammatory antibody drug, adalimumab (Humira). Preliminary SAR analysis of kaempferol analogs showed potential to develop an “Oral Humira” by further improving the potency and efficacy of *Kae* upon structural modification. Moreover, the identification of TNF‐*α* as one of the cellular targets for *MHCl*, a quinolone antibiotic, provides a plausible mechanism accounting for some of its adverse effects. The *n*DEL approach, thus, is undoubtedly a powerful tool for next‐generation pharmaceutical discovery and development.

## 5 Experimental Section

### Cloning, Expression, and Purification

The coding sequences of the extracellular domain of the human TNF‐*α* receptor 1 gene (*h*TNFR1‐ECD, amino acid 22–211 coding sequence from the N‐terminal RDSV to the C‐terminal QIEN), or human TNF‐*α* gene (amino acid 77–233 coding sequence from the N‐terminal VRSS to the C‐terminal IIAL) was PCR amplified from Hela cell cDNA and cloned into a pET‐28a expression vector (Novagen) with an N‐terminal 6 × His tag. The plasmid was transformed into BL21(DE3) cells in a 2 L LB media at 37 °C and 220 rpm, induced with 0.4 mm IPTG after reaching an OD600 (0.8) and expressed at room temperature overnight. The resulting cells were pelleted by centrifugation, and re‐suspended in a 100 mL ice‐cold buffer (20 mm Tris, pH 7.5 with 5 mm EDTA for *h*TNFR1‐ECD; or 25 mm ammonium acetate, pH 6.0, 1 mm DTT, and 1 mm EDTA for *h*TNF‐*α*), and lysed by ultrasonication. The recombinant *h*TNF‐*α* was overexpressed as soluble proteins, whereas *h*TNFR1‐ECD formed inclusion bodies.

Next, primary amines (‐NH2) on *h*TNF‐*α* were biotinylated using the EZ‐LINK NHS‐PEG4‐BIOTIN kit (Thermo, 21330) following the manufacturer's instruction. Briefly, the above recombinant *h*TNF‐*α* was incubated with newly reconstituted sulfo‐N‐hydroxysuccinimide (NHS) esters of biotin (molar ratio of 1:20) at 4 °C for 2 h. Excess biotin was then removed using a desalting column (Thermo Scientific, 89882).

The *h*TNFR1‐ECD inclusion bodies were pelleted twice by centrifugation at 4000 g for 15 min at 4 °C, dissolved in a 40 mL denaturing solution (6 m guanidine‐HCl, 100 mm Tris, pH 8.5, 4 mm PMSF) for 40 min at room temperature, and kept in the denaturing solution supplemented with 20 mm DTT. After 30 min incubation, 30 mm oxidized glutathione was added. After an additional 30 min incubation, the resulting solution was slowly diluted ten‐fold with a buffer (50 mm Tris, pH 10.7, 6 mm cysteine, and 4 mm PMSF), and incubated overnight with gentle stirring at 4 °C. The final solution was centrifuged at 20 000 g for 20 min at 4 °C to remove insoluble debris, dialyzed twice against a 4 L 50 mm Tris buffer (pH 7.5) at 4 °C, and dialyzed twice again with a 4 L buffer (50 mm MES, pH 6.2, 25 mm NaCl) at 4 °C.

The supernatant containing recombinant *h*TNF‐*α* and the renatured *h*TNFR1‐ECD were subject to affinity column purification. For typical affinity purification, insoluble debris was first removed by centrifugation. The supernatant was loaded to a Ni‐NTA agarose (GE) column, rinsed with a washing buffer (25 mm MES, pH 6.2, 25 mm NaCl, and 10 mm imidazole for *h*TNFR1‐ECD; or 25 mm ammonium acetate, pH 6.0, 1 mm DTT and 10 mm imidazole for *h*TNF‐*α*), and eluted with an elution buffer (25 mm MES, pH 6.2, 25 mm NaCl and 300 mm imidazole for *h*TNFR1‐ECD; or 25 mm ammonium acetate, pH 6.0, 1 mm DTT and 300 mm imidazole for *h*TNF‐*α*). The elution fractions containing the desired protein were combined, dialyzed to remove imidazole, concentrated by ultrafiltration, aliquoted, and stored at −80 °C.

The purified *h*TNFR1‐ECD was further conjugated to horse‐radish peroxidase using HRP Conjugation Kit (Proteintech, PK20001) aliquoted, and stored at −20 °C.

### SPR Assay

Binding affinity of small molecule to protein was measured on a BIAcore 8K instrument (GE Healthcare) by SPR assay. For a typical assay, purified *h*TNF‐*α* was dissolved at 30 µg mL^−1^ in an acetate buffer (pH 4.5), and coupled to a CM5 chip (GE Healthcare, 29‐1049‐88) following the manufacturer's instruction. The running buffer (PBS‐P, pH 7.4) contained 1% instead of 5% DMSO in order to keep the activity of *h*TNF‐*α* (Table S2, Supporting Information). Chemicals were 1:2 serial diluted from 50 µm to 1.56 µm (final concentrations) and injected at a flow rate of 30 µL min^−1^ for 90 s for the association step followed by dissociation for an additional 90 s using the LWM multi‐cycle kinetics/affinity method provided by GE Healthcare. Solvent correction was carried out before and after each analysis with eight different concentrations of DMSO solution per cycle. The *K*_D_ value was calculated using Evaluation Software (GE Healthcare).

### Competitive Binding Assay

Microtiter plates (Nunc 96F Maxisorp) were coated overnight with 100 µL/well of 10 µg mL^−1^ avidin (Pierce, 21121) in a 50 mm sodium carbonate buffer (pH 9.0) at 4 °C. After removal of the coating solution, the plates were incubated at 275 µL/well with a blocking solution containing 5% milk v/v in a PBST buffer (pH 7.4) at 37 °C for 1 h. The resulting plates were rinsed once with PBST and incubated at 100 µL/well with a 7.5 nm sparsely biotinylated *h*TNF‐*α* solution (PBS, pH 7.4) for 1 h with shaking. After incubation, plates were rinsed four times with a washing buffer (PBST, pH 7.4).

Testing compounds were 1:10 serial diluted from 100 µm to 1 nm using a solution containing 25 mm MES (pH = 6.2), 25 mm NaCl, 1% DMSO, and 4.6 nm TNFR‐HRP. The final dilution mixture was added to each well of the above plates (100 µL/well) and incubated with shaking for 90 min. Then the plates were washed three times with PBST followed by the addition of 50 µL TMB peroxidase substrate of HRP (Beyotime, P0209‐500 mL) into each well. After incubation at room temperature for 30 min, the reaction was quenched with 2 m sulfuric acid, and absorbance at 450 nm was measured on a microplate reader (Enspire, PerkinElmer).

### Cell Proliferation and Cytotoxicity Assay

The Cell Counting Kit‐8 (CCK8, Meilunbio, MA0218) was applied to measure cell proliferation and survival. For TNF‐*α* induced cell apoptosis measurement, L929 cells that overexpress TNFR1 receptor were seeded at a density of 10^4^ cells/well in a 96‐well microtiter plate and incubated for 24 h in a DMEM media with 10% FBS at 37 °C. The overnight L929 cells were switched into a fresh media containing 0.3 µg mL^−1^ actinomycin D, 1 ng mL^−1^ *h*TNF‐*α*, and/or 10 nm adalimumab (positive control), and/or indicated concentrations of various compounds (in 1% DMSO), and incubated at 37 °C for an additional 20 h. Cell survival was measured by the CCK8 assay (Meilunbio, MA0218). Cells without *h*TNF‐*α* or any testing compound were used as blank, while cells with only *h*TNF‐*α* were used as the negative control.

For cytotoxicity assay of compounds, 10^4^ cells/well L929 cells were seeded in a 96‐well microtiter plate and incubated in a DMEM media with 10% FBS at 37 °C for 24 h. Compounds at different concentrations were next added and incubated for an additional 20 h at 37 °C. Cell survival was measured by CCK8.

### TNF‐*α*‐Induced Signal Transduction in L929

L929 cells were seeded at a density of 10^4^ cells/well in a 96‐well plate and incubated in a DMEM media with 10% FBS at 37 °C for 24 h. The overnight L929 cells were incubated into a fresh DMEM media containing 0.3 µg mL^−1^ actinomycin D, 1 ng mL^−1^ *h*TNF‐*α*, and 50 µm *KB* or *Kae* (in 1% DMSO), or 100 µm *MHCl* (in 1% DMSO), or 20 µm *GCN* (in 1% DMSO), or 10 nm adalimumab (in PBS, pH 7.4), and incubated at 37 °C for an additional 14 h. Cells in each well were lysed, and the cellular proteins were extracted and subject to analysis using BCA Assay Kit (Thermo Scientific, 23227). The amount of *β*‐actin, total Caspase‐3, Cleaved Caspase‐3, and I*κ*B*α* was determined by western‐blot analysis using an anti‐*β*‐actin antibody (Abcam, ab49900), anti‐Caspase‐3 antibody (CST, 9662), anti‐Cleaved Caspase‐3 antibody (CST, 9664), and anti‐I*κ*B*α* antibody (CST, 4814), respectively.

### Acute Irritant Contact Dermatitis Murine Model

All animal experiments were approved by the Institutional Animal Care and Use Committee of ShanghaiTech University. Female C57BL/6 mice (6‐ to 8‐week‐old) were purchased from Shanghai Model Organisms Center. All animals were maintained in a pathogen‐free, temperature‐controlled environment with a 12 h light/dark cycle and provided chow and water ad libitum. All animals were allowed to acclimate for 1 week in the animal facility before handling.

Mice were randomly divided into different cohort study groups. TPA, *Kae*, and indomethacin were dissolved in neat DMSO (vehicle). Atopic treatment (10 µL in volume) was carried out on the inner and outer surfaces of one ear for each mouse. Acute irritant contact dermatitis was induced by 2.4 µg TPA. After 30 min, vehicle, *Indo* (0.5 mg), and *Kae* (0.5 mg) were applied to the TPA treated mouse ear of each cohort group, respectively. After an additional 3 h, a second atopic treatment was carried out to the corresponding mouse ear of each cohort group. After an additional 3 h, mice were sacrificed. Peripheral blood was collected from the canthus vein of mice. Φ 6 mm ear punch biopsies were taken for ear thickness measured with a thickness gauge and for weight. Ear biopsies were stored at −80 °C for the following gene expression experiments.

Biopsies from negative control and treated mice ears in each group were collected and fixed in 4% paraformaldehyde solution overnight at 4 °C. The tissues were embedded in paraffin and then sectioned at a thickness of 6 µm. Tissue sections were stained with hematoxylin and eosin prior to observation with a light microscope.

Peripheral blood was collected from each mouse and treated with red blood cell (RBC) lysis buffer (Biolegend, 420301). Whole cells except erythrocytes were collected by centrifuged at 500 g for 10 min and incubated with anti‐Mo Ly‐6G/Ly‐6C (Invitrogen, 11‐5931‐82) antibody for 20 min. Cells were washed several times and analyzed on a flow cytometer (CytoFLEX, Beckman Coulter).

The above ear biopsies were lysed by TRIzol Reagent (Invitrogen) regent and homogenized by a homogenizer (JXSFTPRP‐48, Shanghai Jing Xin). Total RNA was obtained from lysed cells and then reverse‐transcribed into cDNA using the Vazyme kit (R212‐02) following the manufacturer's protocol. RT‐qPCR was performed in triplicates with 2 × SYBR Green qPCR Master Mix (Bimake, B21202) on CFX384 Real‐Time System (BIO‐RAD). Messenger RNA expression levels of TNF‐*α*, IL‐1*β*, and CXCL2 were measured and normalized to the internal control genes (*β*‐actin). The primer sequences are listed in Table S2, Supporting Information.

### Statistical Analysis

Experiments were repeated at least three times, and the results were expressed as means ± standard deviation (S.D.) unless otherwise indicated. Sample numbers are indicated in figure legends. Data analysis was performed with GraphPad Prism software. Four parameter non‐linear regression was used for fitting and *IC*_50_ calculation. Significance was assumed at a *p*‐value <0.05 by using a two‐tailed Student's *t*‐test or one‐way ANOVA for comparisons of two groups or more than two groups, respectively, in the software for normally distributed data sets with equal variances, (* *p* < 0.05, very significant for ** *p* < 0.01, and the most significant for *** *p* < 0.001).

## Conflict of Interest

The authors declare no conflict of interest.

## Author Contributions

S.W., X.S., and J.L. contributed equally to this work. Prof. Richard A. Lerner directed and provided insight and method to this study, a work he viewed highly for DEL application before his passing away. S.W. performed the selection, the biochemical experiments, in vivo experiments, and analyzed the data. J.L. synthesized the DEL library and contributed to the SPR experiments. Q.H., X.S., Q.J., L.L., Y.Y., and Y.D. helped in the experiments. X.S. and T.W. helped in the in vivo experiments. M.Y. provided the GCN chemicals. S.W., P.M., H.X., and J.L. analyzed the data. P.M., H.X., and G.Y. designed the study and wrote the manuscript. All authors read and approved the final manuscript.

## Supporting information

Supporting Information

Suplemental Table 4

## Data Availability

The data that support the findings of this study are available from the corresponding author upon reasonable request.
