# Supplementary material for: A Small Molecule Selected from a DNA‐Encoded Library of Natural Products That Binds to TNF‐α and Attenuates Inflammation In Vivo
Source: Adv Sci (Weinh). 2022 May 21;9(21):2201258. doi: 10.1002/advs.202201258 (PMC9313502; doi:10.1002/advs.202201258)
Supplement: Supplementary file 1 — Supporting Information [file ADVS-9-2201258-s001.pdf]

Supporting Information

A Small Molecule Selected from a DNA-Encoded Library of Natural Products that Binds to TNF-α and Attenuates Inflammation in vivo

- In Memory of Professor Richard A. Lerner -

Shuyue Wang†, Xiaojie Shi†, Jie Li†, Qianping Huang, Qun Ji, Ying Yao, Tao Wang, Lili Liu, Min Ye, Yun Deng, Peixiang Ma, Hongtao Xu*, Guang Yang**

Supplementary Figures and Tables:

Contents

Figure S1 2
Figure S2 3
Figure S3 4
Figure S4 5
Figure S5 6
Figure S7 8
Figure S8 9
Table S1 10
Table S2 11
Table S3 12
Supplementary experimental methods 13
References 15

Figure S1

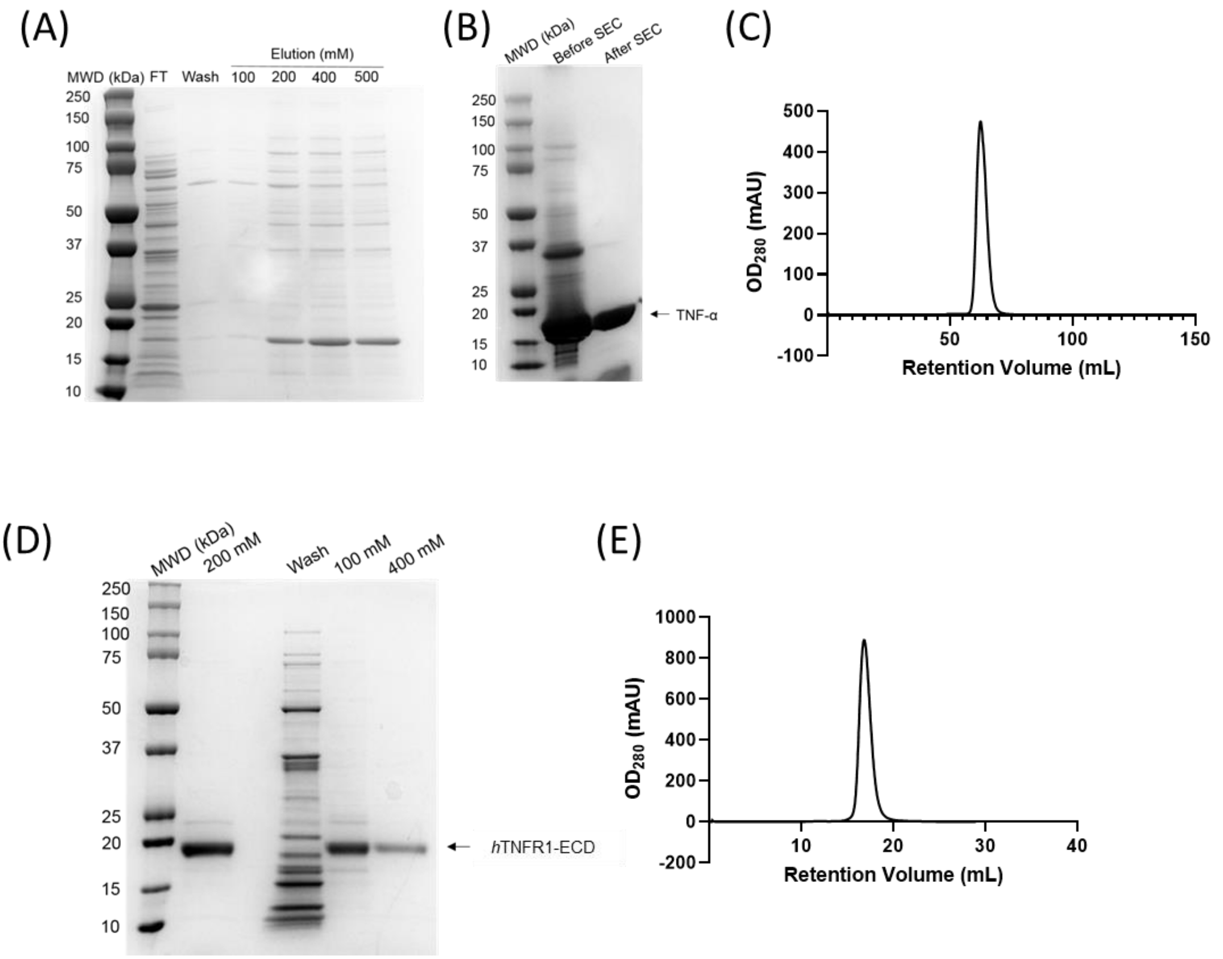

Figure S1. Expression and Purification of recombinant human TNF-α (*h*TNF-α) and human TNFR1 extracellular domain (*h*TNFR1-ECD). (**A**) SDS-PAGE analysis of the Ni-NTA affinity purified *h*TNF-α. (**B**) SDS-PAGE analysis of the purified TNF-α after SEC. (**C**) Size-exclusion-chromatography (SEC) purification profile of *h*TNF-α. (**D**) SDS-PAGE analysis of *h*TNFR1-ECD affinity purification using Ni-NTA . (**E**) SEC purification profile of *h*TNFR1-ECD.

Figure S2

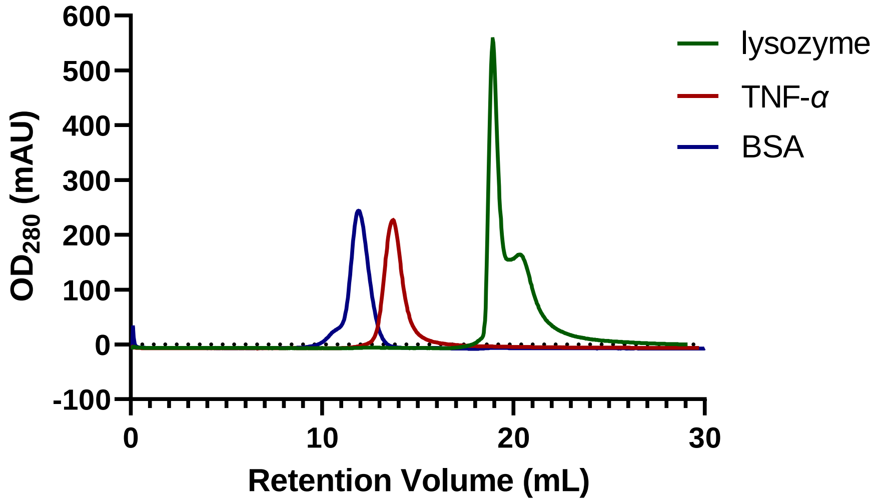

Figure S2. Recombinant *h*TNF-*α* was analyzed using the size exclusion chromatography (SEC). The MW of trimeric his-tagged *h*TNF-*α* is 55.2 kDa. *h*TNF-*α* was eluted with similar volume as BSA (MW. 66.4 kDa), but not appear in the monomer position, similar to Lysozyme (MW. 17.9 kDa).

Figure S3

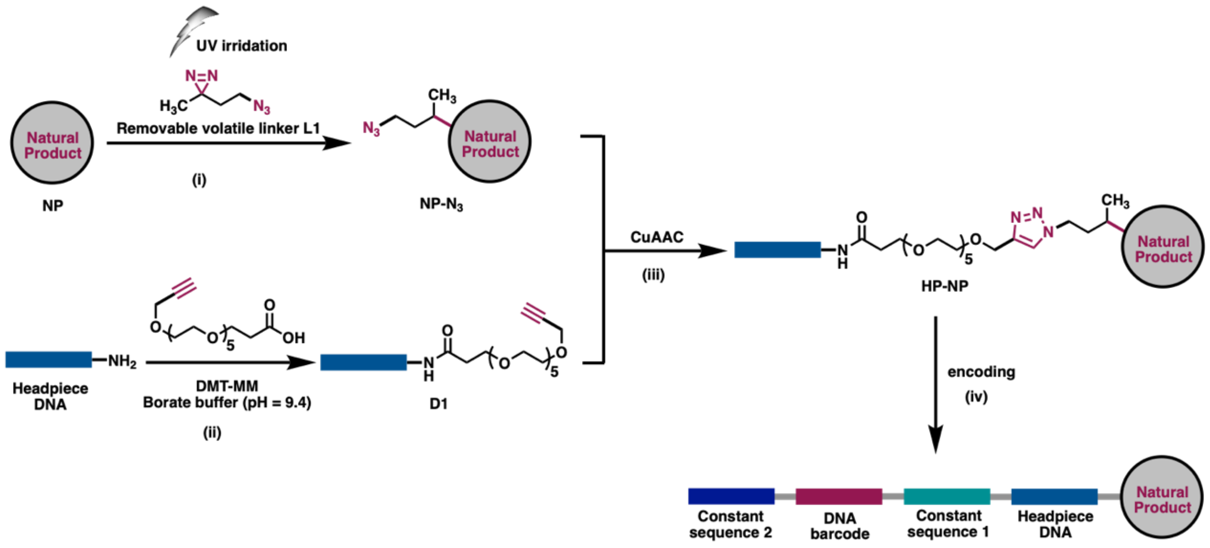

Figure S3. General Procedure for the functionality-independent DNA-encoding of natural products**.** The method that used for DNA annotation of natural products in 96 well plate was according to previous work (*Angew. Chem. Int. Ed*. 2019 (58), 9254-9261). Briefly, natural products were dissolved in organic solvent or water and added into each well in 96-well plate, and mixed with 5 equivalents of bi-functional linker **L1** (dissolved in CH_3CN). Then the chemo- and site nonselective reaction was initiated by UV irradiation at 365 nm (*step i*). After completion of the reaction, side products of linker **L1** and/or the remaining **L1** can be readily removed under vacuum. After LC-MS analysis of L1 labelled natural products, we conjugate alkyne modified headpiece DNA with **L1**-labelled natural products by CuAAC (*step iii*), and subsequent barcoding by enzymatic DNA ligation (*step iv*). Finally, the final product of each well was pooled together to generate a DNA-encoded natural product library (*n*DEL). The content of the *n*DEL has been listed in **Table S4**, Supporting Information.

Figure S4

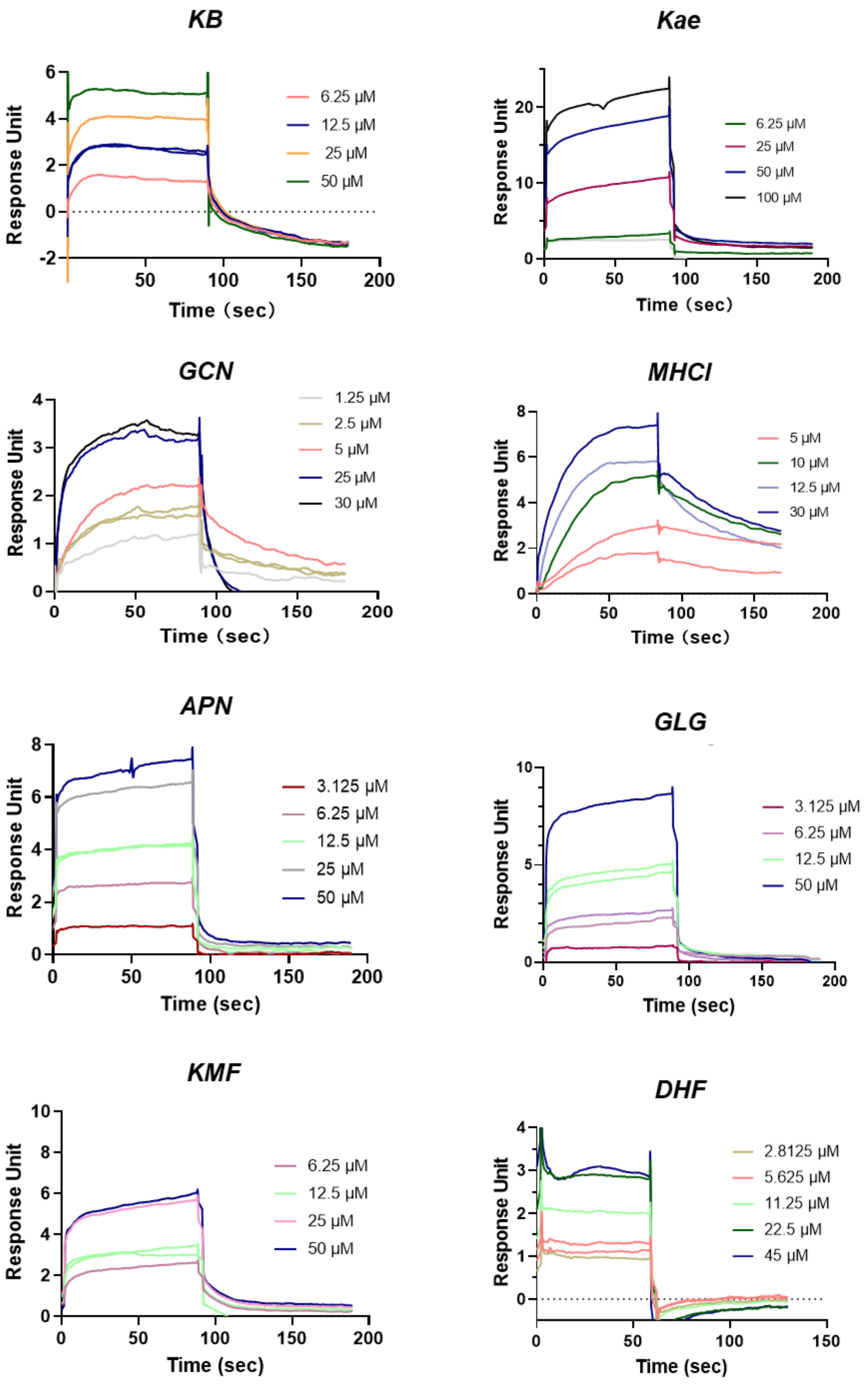

Figure S4. Original SPR sensorgram traces for each corresponding *n*DEL hit.

Figure S5

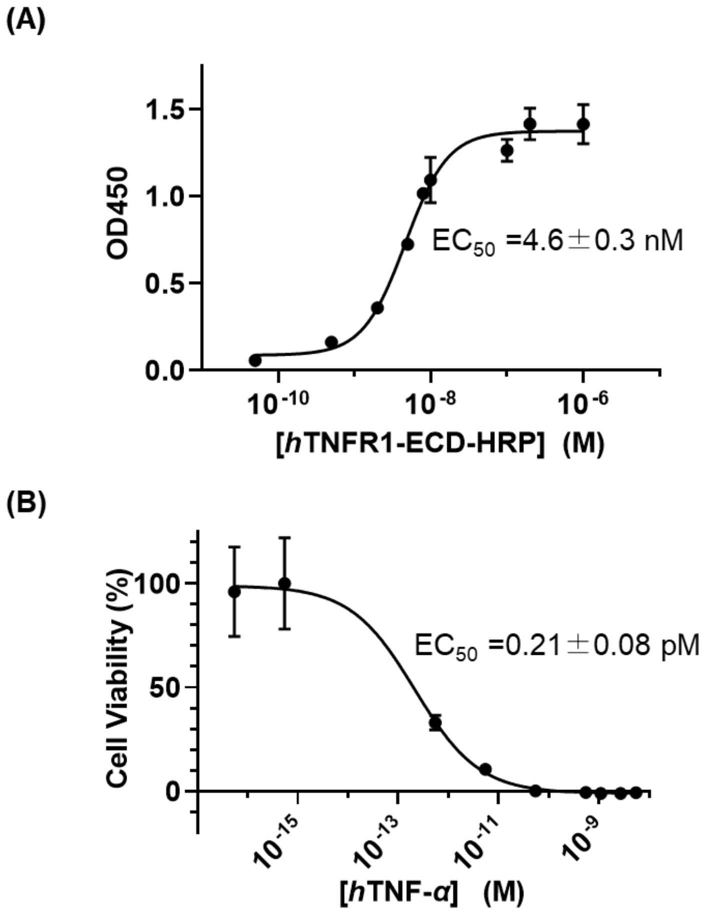

Figure S5 (A) Binding titration between *h*TNF-*α* and *h*TNFR1-ECD-HRP; **(B)** *h*TNF-*α* induced cell death of L929 cell line. Results are shown as mean ± S.D. (*n*=4).

Figure S6

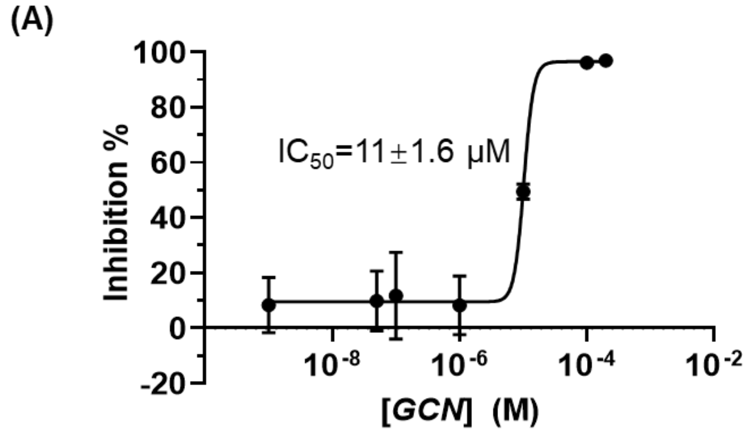

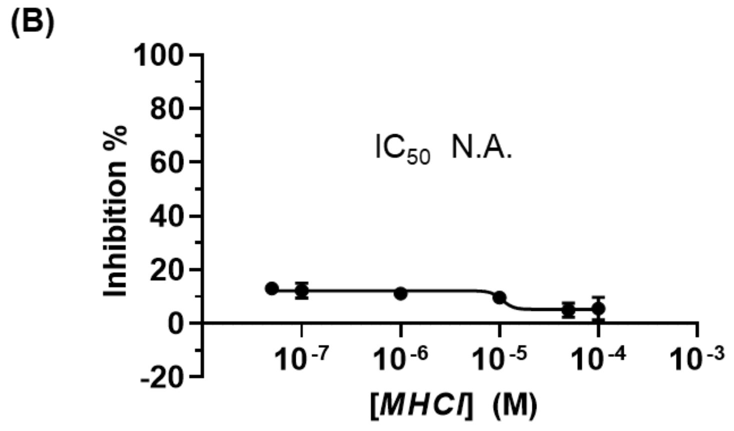

Figure S6. Competition assay of *GCN* and *MHCl* binding to *h*TNF-*α*. **(A)** Competition assay of *GCN* binding to *h*TNF-*α* in the presence of 4.6 nM *h*TNFR1-ECD-HRP; **(B)** Competition assay of *MHCl* binding to *h*TNF-*α* in the presence of 4.6 nM *h*TNFR1-ECD-HRP. Results are shown as mean ± S.D. (*n*=4).

Figure S7

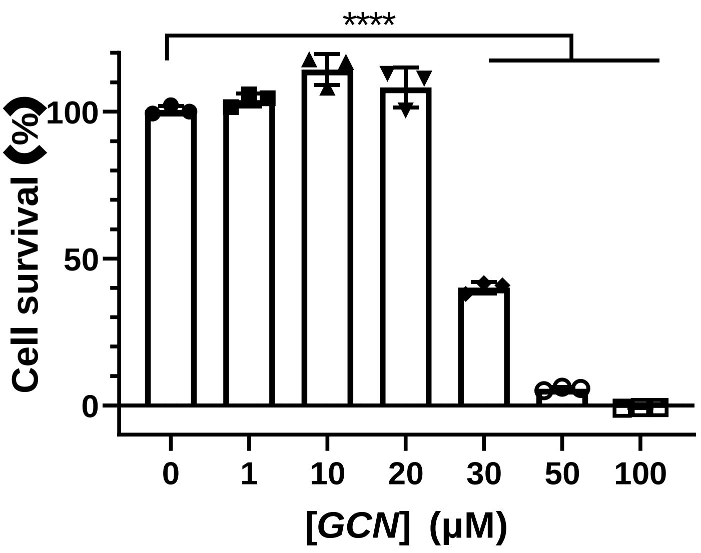

Figure S7. *GCN* induced cytotoxicity in L929 cell line. Experiment was carried out in triplicates and results are shown as means ± S.D. (*n* = 3). One-way ANOVA with multiple-comparisons was carried out, ****P < 0.0001.

Figure S8

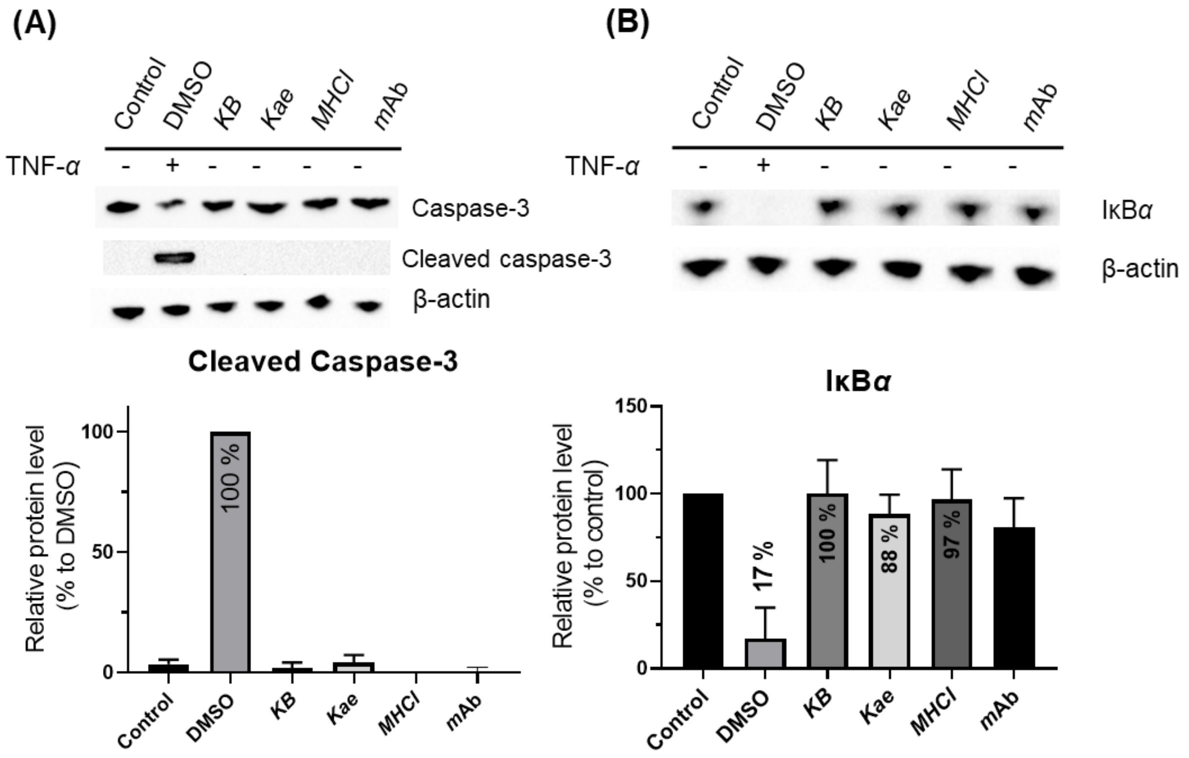

Figure S8. Effects of compounds (*KB, Kae, MHCl*) or Adalimumab (*m*Ab) alone without TNF-*α* stimulation on **(A)** caspase-3 activation; and **(B)** IκB*α* degradation in L929 cells. Upper panels: representative western-blots; Lower panels: relative cellular proteins of cleaved caspase-3 and IκB*α*. Results are shown as means ± S.D. (*n*=3).

Table S1

Table S1. Comparison of enrichment-fold *vs.* affinity for confirmed *n*DEL hits.

**Chemical**	**Enrichment fold**	**Affinity (K_D) [μM]**
Flumequine (*FMQ*)	358	+
Moxifloxacin hydrochloride (*MHCl*)	102	7.8 ± 1.5
Gancaonin I (*GCN*)	69	1.9 ± 0.2
Semilicoisoflavone B (*SFB*)	57	+
Kumatakenin B (*KB*)	46	15 ± 5.8
Kaempferol (*Kae*)	45	46 ± 5.5
Gentisic acid (*GA*)	34	+
Glycyrol (*GCR*)	34	+

Table S2

Table S2. Thermal stability (T_m) of recombinant *h*TNF-*α* in various %DMSO

**Samples**	**T_m [□]**
TNF-*α* 0% DMSO	73.1
TNF-*α* 1% DMSO	71.2
TNF-*α* 2% DMSO	69.5
TNF-*α* 3% DMSO	66.7
TNF-*α* 4% DMSO	65.2

Table S3

Table S3. Primers for the qPCR detection of cytokine expression.

**Oligo names**	**Sequences**
TNF-α Forward	5'-CATCTTCTAAAATTCGAGTGACAA-3'
TNF-α Reverse	5'-TGGGAGTAGACAAGGTACAACCC-3'
IL-1β Forward	5'- GAAGAAGAGCCCATCCTCTG-3'
IL-1β Reverse	5'- TCATCTCGGAGCCTGTAGTG -3'
CXCL2 Forward	5'- CCCCCTGGTTCAGAAAATCATC -3'
CXCL2 Reverse	5'- AACTCTCAGACAGCGAGG CACATC -3'

Supplementary experimental methods

General Procedure for the functionality-independent DNA-encoding of natural products

The synthetic scheme for DNA annotation of natural products in a 96-well plate was according to previous work (*Angew. Chem. Int. Ed.* 2019 (58), 9254-9261). Briefly, natural products were dissolved in organic solvent or water and added into each well in 96-well plate, and mixed with 5 equivalents of bi-functional linker **L1** (dissolved in CH_3CN). Then the chemo- and site nonselective reaction was initiated by UV irradiation at 365 nm (*step i*). After completion of the reaction, side products of linker **L1** and/or the remaining **L1** could be readily removed under vacuum. After LC-MS analysis of L1 labelled natural products, we conjugated alkyne modified headpiece DNA with **L1**-labelled natural products by CuAAC (*step iii*), and subsequent barcoding by enzymatic DNA ligation (*step iv*). Finally, the final product of each well was pooled together to generate a DNA-encoded natural product library (*n*DEL). The content of the *n*DEL is listed in **Table S4**, Supporting Information.

Size Exclusion Chromatography (SEC) of *h*TNF-*α* (for Figure S2)

The oligomeric state of recombinant *h*TNF-*α* was subject to analytical size exclusion chromatography analysis as previously described.[1] A running buffer containing 10 mM Tris pH 7.5, 50 mM NaCl, and 1 mg recombinant *h*TNF-*α* was loaded onto a Superdex 75 10/300 GL column. 1 mg BSA (66.4 kD) and 1.5 mg lysozyme (17.9 kD) in the same buffer were injected onto the same Superdex column as molecular weight marker controls.

Selection of hits from the *n*DEL

The recombinant *h*TNF-α was biotinylated via lysine conjugation using the EZ-Link NHS-PEG4-Biotin kit (Thermo Fisher Scientific, # A39259). The biotinylated protein was used to bind to streptavidin-coated Dynabeads M280 (Thermo Fisher Scientific, # 11205D). The Dynabeads were washed twice using PBST in 5 minute intervals. The beads were separated using a magnetic rack to separate from the supernatant. For each screening, 10 μg biotinylated-*h*TNF-*α* was first immobilized on 12 μL streptavidin-coated Dynabeads M280 (Thermo Fisher Scientific, #11205D) with 200 μg/mL Salmon sperm DNA (Thermo Fisher Scientific, #15632-011) as the blocking buffer, by incubation at room temperature for 1 hour

in a total volume of 50 μL. Negative control was set with biotinylated-*h*TNF-*α* replaced by PBS.

After two washes of the immobilized target and the negative control with PBST, *n*DEL was added to them. The final volume of the selection system is 100 μL, with concentration of each DNA-encoded chemicals *around* 0.05 pM. The selection system was incubated at room temperature with rotation for 1 hour. Following five washes with PBST, *h*TNF-*α* bound chemical-DNA conjugates were eluted from the target by heating at 95 ℃ for 10 min in 90 μL PBST.

Subsequent second and third rounds of selection were performed by incubating the eluant from the previous round with the bead-immobilized *h*TNF-*α* in the same condition except for the third round final elution by incubation with 10 μM SPD304 at room temperature for 30 min instead of heating. The resulting final elution was collected for sequencing analyses.

DNA sequencing and data analyses

DEL decoding was performed as previously described[2]. In brief, DNA tags were amplified by PCR using the the following primers:

Forward:5’-AATGATACGGCGACCACCGAGATCTACACTCTTTCCCTACACG

Reverse:5’-CAAGCAGAAGACGGCATACGAGATGTCGTGATGTGACTGGAGTTC.

Universal primers were appended using the sequencing primer designed for the Illumina platform. Totally three types of samples were sequenced, *i.e.* naïve library reference for normalization, screened pool against *h*TNF-*α* and screened pool of negative control. The Illumina adaptor sequences in the PCR amplification were trimmed using CLC genomics workbench version 12 (Qiagen). After adaptor trimming and length filtering, a total of 45,228,755 reads, 20,749,217 reads and 31,290,087 reads were collected for the subsequent analysis for the three samples, respectively. For each testing sample, the trimmed data were mapped to the reference library. No mismatch was allowed in the mapping procedure. The mapping ratio of the three samples to the designed bar-coding sequences are 90.3%, 50.5% and 63.6%, respectively. Read count of each sequence corresponding to a distinct chemical was normalized to that of the reference sample in order to calculate the enrichment fold. The mapped coding sequences were counted for all chemicals across different samples. The total sequencing counts (S_{total}) represent the coding sequences counted for all chemicals in a given sample. The sequencing counts (S) for each individual chemical were normalized using the following equation (eq. 1), in which S_0 represents the normalized S.

$$S_0 = 100{,}000 \times S / S_{total} \text{ (eq. 1)}$$

An in-house java program was developed to analyze enrichment of *n*DELs during the screening. The fold changes of normalized sequencing counts (*i.e.* enrichment fold) for each chemical in *n*DEL after incubation with target protein were calculated in comparison with that in the reference library as shown in the equation below:

$$\text{enrichment fold} = S_{0,\ sample} / S_{0,\ reference} \text{ (eq. 2).}$$

Finally, the enrichment fold against the sequence count were plotted as the panning fingerprints (Figure 1B).

Protein thermal stability assay (for Table S2)

The recombinant *h*TNF-α was diluted in a PBS-P buffer containing no DMSO, 1% DMSO, 2% DMSO, 3% DMSO and 4% DMSO. Samples were subject to nano-DSF analyses on The Prometheus NT.48 (NanoTemper Technologies GmbH). Nano-DSF grade capillaries and capillary chips were automatically filled with the corresponding samples by capillary forces when dipping into different sample solutions. The rate of thermal ramp-up from 20 °C to 95 °C across capillary tray was set up at 1 °C mL^{-1}. Thermal unfolding transition temperatures (T_m) of *h*TNF-α were determined by fluorescence intensity changes at 330 nm and 350 nm.

EC_{50} of *h*TNF-α binding to *h*TNFR-HRP by ELISA

Biotinylated *h*TNF-α was immobilized on a microtiter plate (Nunc 96F Maxisorp) as same as that for the *h*TNFR competition assay described in Experimental section. Serial dilutions of *h*TNFR-HRP from 1 μM to 50 pM in PBS were added to each *h*TNF-α coated well and incubated at room temperature with shaking for 90 min. Plates were then washed 3 times with PBST followed by the addition of 50 μL TMB peroxidase substrate of HRP (Beyotime, P0209-500 mL) into each well. After incubation at room temperature for 30 min, peroxidase reaction was quenched with 2 M sulfuric acid. Absorbance at 450 nm was measured on a microplate reader (Enspire, PerkinElmer). EC_{50} was calculated by non-linear regression fit using Prism.

References

[1] L. C. Santora, Z. Kaymakcalan, P. Sakorafas, I. S. Krull, K. Grant, Anal Biochem **2001**, 299, 119.

[2] J. Xie, S. Y. Wang, P. X. Ma, F. Ma, J. Li, W. Wang, F. P. Lu, H. Xiong, Y. Gu, S. N. Zhang, H. T. Xu, G. Yang, R. A. Lerner, iScience **2020**, 23.
